# To TRIM the Immunity: From Innate to Adaptive Immunity

**DOI:** 10.3389/fimmu.2020.02157

**Published:** 2020-10-08

**Authors:** Wenyong Yang, Zhiwen Gu, Huiyuan Zhang, Hongbo Hu

**Affiliations:** Department of Rheumatology and Immunology, National Clinical Research Center for Geriatrics, and State Key Laboratory of Biotherapy, West China Hospital, and West China School of Basic Medical Sciences & Forensic Medicine, Sichuan University, and Collaborative Innovation Center for Biotherapy, Chengdu, China

**Keywords:** tripartite motif, innate immunity, adaptive immunity, ubiquitination, signal

## Abstract

The tripartite motif (TRIM) proteins have been intensively studied as essential modulators in various biological processes, especially in regulating a wide range of signaling pathways involved in immune responses. Most TRIM proteins have E3 ubiquitin ligase activity, mediating polyubiquitination of target proteins. Emerging evidence demonstrates that TRIM proteins play important roles in innate immunity by regulating pattern recognition receptors, vital adaptor proteins, kinases, and transcription factors in innate immune signaling pathways. Additionally, the critical roles of TRIM proteins in adaptive immunity, especially in T cell development and activation, are increasingly appreciated. In this review, we aim to summarize the studies on TRIMs in both innate and adaptive immunity, focusing on their E3 ubiquitin ligase functions in pattern recognition receptor signaling pathways and T cell functions, shedding light on the developing new strategies for modulating innate and adaptive immune responses against invading pathogens and avoiding autoimmunity.

## Introduction

The immune system, consisting of innate and adaptive immune arms, provides crucial protection for the host against infectious agents and serves as a housekeeper for immune homeostasis. The innate immune system is the first line of defense against various pathogens. Using a series of germline-encoded pattern recognition receptors (PRRs), including Toll-like receptors (TLRs), NOD-like receptors (NLRs), RIG-I-like receptors (RLRs), C-type lectin receptors (CLRs), and cytosolic DNA-sensing receptors, innate immune cells recognize both exogenous pathogen-associated molecular patterns (PAMPs) and endogenous damage-associated molecular patterns (DAMPs) (1–6). The adaptive immune system, on the contrary, provides the more sophisticated immune response mediated by T and B lymphocytes, which are armed by highly specific antigen-recognizing receptors, T cell receptors (TCR) and B cell receptors (BCR), respectively. The immune system is tightly controlled by signaling networks in all types of immune cells in a spatial-temporal manner to launch an appropriate immune response against infections without causing tissue damage. The dynamic regulation of these signaling networks relies greatly upon various protein posttranslational modifications, highlighting their irredundant roles in immune responses.

Ubiquitination is one of the major protein posttranslational modification mechanisms in eukaryotes, regulating various cellular procedures, such as DNA damage repair, cell cycle regulation, and signal transduction, which is mediated by conjugating mono- or polyubiquitin chains to the lysine (K) residues of target proteins (7–10). Ubiquitination is mediated by a three-enzyme system: ubiquitin-activating enzymes (E1), ubiquitin-conjugating enzymes (E2, also named ubiquitin-carrier protein), and ubiquitin ligases (E3). The E3 ubiquitin ligases (more than 700 in the human genome) are responsible for the substrate specificity (11–13). The conjugated ubiquitin can be reversibly cleaved by deubiquitinases (DUBs), making ubiquitination a reversible and dynamic procedure (10, 14). Currently, the crucial roles of ubiquitination in the regulation of immune responses have been intensively studied.

**Figure 1:**
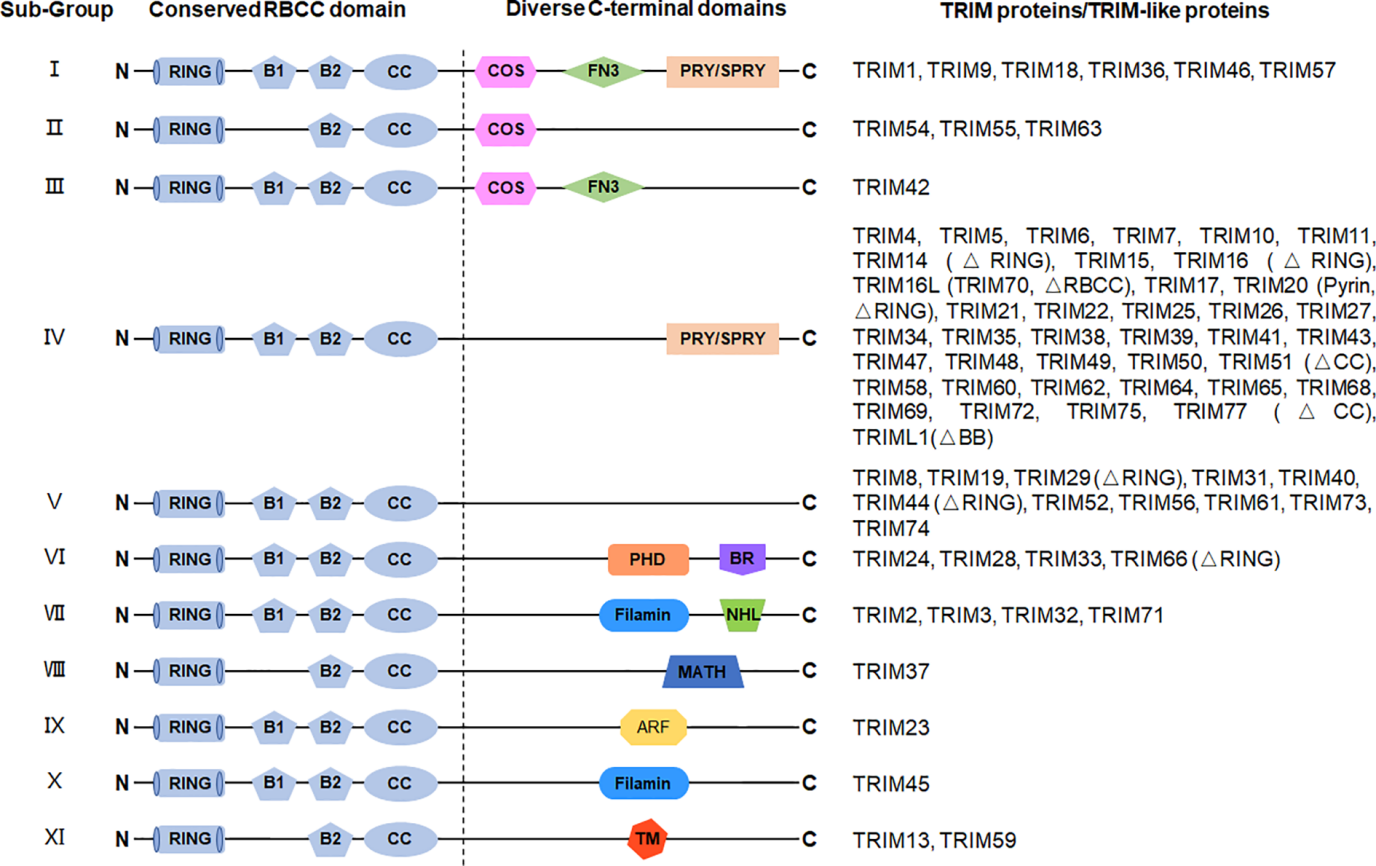
Structures of TRIM protein. TRIM proteins are generally divided into 11 subgroups depending on their variable C-terminal domains, including C-terminal subgroup one signature (COS) domain, fibronectin type 3 (FN3) domain, SPIa and the ryanodine receptor (SPRY) domain, SPRY-associated domain (PRY), plant homeodomain (PHD), bromodomain (BR), filamin domain, NHL repeats (NHL) domain, meprin and tumor necrosis factor receptor–associated factor homology (MATH) domain, ADP-ribosylation factor (ARF) domain, and transmembrane (TM) domain. The N-terminal domains are conserved among TRIM proteins, containing a RING domain, a B-box 1 (B1) and/or a B-box 2 (B2) domain, and a CC domain. TRIM-like proteins lacking typical domain(s) are indicated in brackets.

Tripartite motif (TRIM) proteins are a predominant family of the E3 ubiquitin ligases with more than than 70 members in humans and characterized by the conserved N-terminal tripartite RBCC motif, which contains a really interesting new gene (RING) domain, one or two B-BOX domain(s), followed by a coiled-coil domain (**Figure 1**) (15, 16). The expansion of the *TRIM* multigene family is strikingly paralleled with the evolution of innate and adaptive immunity, suggesting that TRIM proteins may function as regulatory factors for increasingly complicated immune functions (17, 18). Consistently, a line of studies has shown that the expression of TRIM responds to various stimuli, for example, IFNs, LPS, and virus components (19–24). Emerging evidence has indicated that TRIM proteins play crucial roles in regulating host defense against pathogens and the pathogenesis of autoimmune diseases. In this review, we focus on the current progress in understanding the roles of TRIM proteins in both innate and adaptive immune responses.

## Structure of TRIM Proteins

The name “TRIM proteins” comes from the highly conserved RBCC motif, standing for the RING–BBox–CC motif in their N-terminals (**Figure 1**) (15, 16). The RING domain contains the Cys-His-rich domain, which binds to two zinc atoms and presents in almost all TRIM proteins (25). The RING domain exerts ubiquitin E3 ligase activity by providing a docking site for E2 conjugases and promoting ubiquitin ligation, which endows TRIM proteins with potential ubiquitin E3 ligase activity. Indeed, a large portion of TRIM proteins have been identified as ubiquitin E3 ligases to mediate different types of ubiquitination of diverse substrates (**Table 1**).

**Table 1:** Regulation of immune responses by TRIM proteins and their regulatory functions.

TRIM	Target molecule	Function	Refs
**TLR-mediated innate immune response**			
**TRIM38**	TRIF	Promoting proteasomal degradation of TRIF	(26, 27)
**TRIM56**	TRIF	Interacting with TRIF physically to promote TLR3 signaling activation	(28)
**TRIM23**	NEMO	Mediating K27-linked ubiquitination of NEMO	(29)
**TRIM23**	TRAF6	Promoting TRAF6 autoubiquitination	(30)
**TRIM29**	NEMO	Inducing proteasomal degradation of NEMO	(31)
**TRIM27**	IKKs	Interacting with the noncanonical and canonical IKK family members	(32)
**TRIM28 (KAP1)**	IRF7	Mediating SUMOylation of IRF7 to suppress transactivation	(33)
**TRIM21 (Ro52/SS-A)**	IRF7	Inducing proteasomal degradation of IRF7	(34)
**TRIM21**	IRF3	Inducing proteasomal degradation of IRF3	(35)
**TRIM21**	IRF3	Mediating autophagic degradation of IRF3	(36, 37)
**TRIM19IV**	Pin1	Regulating the cellular distribution of Pin1	(38, 39)
**TRIM38**	TRAF6	Inducing proteasomal degradation of TRAF6	(40)
**TRIM12c**	TRAF6	Interacting with TRAF6 and inducing TRAF6 ubiquitination	(41)
**TRIM13**	TRAF6	Inducing TRAF6 ubiquitination	(42)
**TRIM5α**	TAK1	Generating free ubiquitin chains, which activate the TAK1 kinase	(43, 44)
**TRIM30α**	TAB2 and TAB3	Interacting with TAK1 complex and promoting TAB2 and TAB3 degradation	(45)
**TRIM22**	TAB2	Targeting TAB2 for degradation	(46)
**TRIM21**	TAK1	Generating free ubiquitin chains, which activate the TAK1 kinase	(47, 48)
**TRIM40**	NEMO	Mediating neddylation of NEMO	(49)
**TRIM21**	IKKβ	Catalyzing monoubiquitin of IKKβ, which leads to IKKβ degradation by autophagolysosome	(50, 51)
**TRIM19**	RelA/p65	Interacting with RelA/p65	(52)
**TRIM9**	β-TrCP	Interacting with β-TrCP and stabilizing IκBα and p100	(53)
**TRIM20 (Pyrin)**	IκBα and p65	Interacting with IκBα and p65 to promote IκBα degradation and p65 translocation into the nucleus by the PYD domain	(54)
**TRIMs function in RLR-mediated innate immune response**			
**TRIM25**	RIG-I	Mediating K63-ubiquitination of RIG-I	(55, 56)
**TRIM25**	MAVS	Promoting MAVS ubiquitination to release the signaling complex into cytosol	(57)
**TRIM4**	RIG-I	Mediating K63-ubiquitination of RIG-I	(58)
**TRIM65**	MDA5	Promoting K63-ubiquitination of MDA5	(59, 60)
**TRIM13**	MDA5	Interacting with MDA5 and negatively regulating type I interferon production	(61)
**TRIM14**	MAVS	Interacting with MAVS and promoting the recruitment of NEMO	(62)
**TRIM44**	MAVS	Interacting with MAVS and stabilizing MAVS	(63)
**TRIM31**	MAVS	Promoting K63-polyubiquitination and aggregation of MAVS	(64)
**TRIM9s**	TBK1	Promoting phosphorylation of TBK1	(65)
**TRIM38**	NAP1	Inducing proteasomal degradation of NAP1	(66)
**TRIM26**	NEMO	linking TBK1 to NEMO for TBK1 activation	(67)
**TRIM26**	IRF3	Inducing proteasomal degradation of IRF3	(68)
**TRIM11**	TBK1	Interacting with TBK1 and inhibiting its activation	(69)
**TRIM68**	TFG	Inducing lysosomal degradation of TFG	(70)
**TRIMs function in cytosolic DNA-sensing receptors-mediated innate immune response**			
**TRIM21**	DDX41	Inducing proteasomal degradation of DDX41	(71, 72)
**TRIM14**	cGAS	Inhibiting the degradation of cGAS	(73)
**TRIM38**	cGAS	Mediating SUMOylation of cGAS to inhibit cGAS degradation	(74)
**TRIM38**	STING	Mediating SUMOylation of STING to inhibit STING degradation	(74)
**TRIM56**	STING	Promoting K63-linked ubiquitination of STING	(75)
**TRIM32**	STING	Promoting K63-linked ubiquitination of STING	(76)
**TRIM30α**	STING	Inducing proteasomal degradation of STING	(77)
**TRIMs function in NLR-mediated inflammasome signaling**			
**TRIM20**	NLRP3, Caspase-1, and Pro-IL-1β	Interacting with NLRP3, caspase-1, and Pro-IL-1β to inhibit caspase-1 activation and IL-1β secretion	(78–80)
**TRIM20**	ASC	Interacting with ASC to promote inflammasome activation	(81)
**TRIM16 (EBBP)**	NLRP1, Caspase-1 pro-IL-1β	Interacting with NLRP1, caspase-1 and pro-IL-1β to promote IL-1β secretion	(82)
**TRIM30**	NLRP3	Increasing ROS production	(83)
**TRIM31**	NLRP3	Inducing proteasomal degradation of NLRP3	(84)
**TRIM33**	DHX33	Promoting K63-ubiquitination of DHX33 to form DHX33-NLRP3 signalosome	(85)
**TRIM27**	NOD2	Inducing proteasomal degradation of NOD2	(86)
**TRIM11**	AIM2	Degrading AIM2 *via* selective autophagy	(87)
**TRIMs function in T cell signaling**			
**TRIM27**	PI3KC2β	Promoting K48-ubiquitination of PI3KC2β to suppress the functions of CD4 T cells	(88)
**TRIM30**	Not described	Promoting T cell activation but inhibiting T cell proliferation	(89)
**TRIM24**	Not described	Promoting IL-1R expression on Th2 cells	(90)
**TRIM32**	Not described	Impairing Th2 biased response	(91)
**TRIM21**	Not described	Promoting CD28-mediated IL-2 production	(92)
**TRIM21**	Not described	Negatively regulating the IL-23-Th17 pathway	(93)
**TRIM33**	Not described	Participate in the TGF-β signaling cascades	(94, 95)
**TRIM28**	Not described	Preventing autoinflammatory T cell development	(96)
**TRIM28**	FIK and Foxp3	Suppressing Foxp3 transcriptional activity and regulating Treg function	(97)
**TRIM28**	TCRα locus	Promoting the development of T and iNKT cells	(98)
**TNFα and IL-1β-mediated signaling**			
**TRIM8**	TAK1	Promoting K63-linked polyubiquitination of TAK1 for activation	(99)
**TRIM38**	TAB2/3	Mediating lysosomal degradation of TAB2/TAB3	(100)
**TRIM13**	NEMO	Mediating ubiquitination and turnover of NEMO to suppress NF-κB activation	(101)
**TRIM45**	Not described	Negatively regulating NF-κB activation	(102)
**Other**			
**TRIM18 (midline 1)**	PP2A	Inhibiting protein phosphatase 2A (PP2A) activity	(103)
**TRIM6**	IKKϵ	Promoting unanchored K48-linked polyubiquitin of IKKϵ and leading to IKKϵ activation	(104)

The B-Box domain is very similar to the RING domain and identified as a zinc-finger domain, which is found exclusively in TRIM proteins, except for NRDP1/RNF41. The B2-Box domain exists in all TRIM proteins although the B1-Box domain is only found in certain subfamilies, which differs from the B2-Box domain in the different spacing of zinc-binding residues. Given the similarity of the two B-Box domains and the RING domain in the three-dimensional structure, these three domains might be derived from a common ancestor (105). The function of B-Box is currently poorly understood. Recent studies on TRIM proteins indicate that the B-Box domain contributes to the innate immune response against HIV infection (106–108). For TRIM proteins without the RING domain, the B-Box domain could exert the activity of E3 ubiquitin ligase by providing a binding site for E2 ubiquitin-conjugating enzymes, which is similar to that of the RING domain (18, 31, 109, 110). However, the functions of the B-Box domain in the RING-containing TRIM proteins remain unclear. Whether the B-Box domain is dispensable or it collaborates with the RING domain for the E3 ligase activity of TRIM proteins needs to be further studied. The coiled-coil (CC) domain is a common protein helical region with a variety of biological functions. The CC domain in TRIM proteins is required and sufficient for homo- or hetero-oligomerization of TRIM proteins (111, 112). These different oligomerizations endow the TRIM proteins with various biological functions. It is also suggested that the CC domain might be necessary for the formation of subcellular structures (for instance, PML/TRIM19 nuclear bodies) (113, 114).

The C-terminal domains of TRIM proteins are diverse (**Figure 1**) (16, 115), which is correlated to the diversity of the biological functions of TRIM proteins through specific recruitment of interacting proteins. Based on different C-terminal structures, TRIM proteins are further divided into 11 subfamilies. The most common one is the PRY-SPRY domain (also known as the B30.2 domain), covering more than half of the TRIMs (116). Among the large family of TRIM proteins, many TRIMs are characterized for their pivotal roles in the immune response, especially in the innate immune response against viral infections.

## TRIMs Function In TLR-Mediated Innate Immune Response

**Figure 2:**
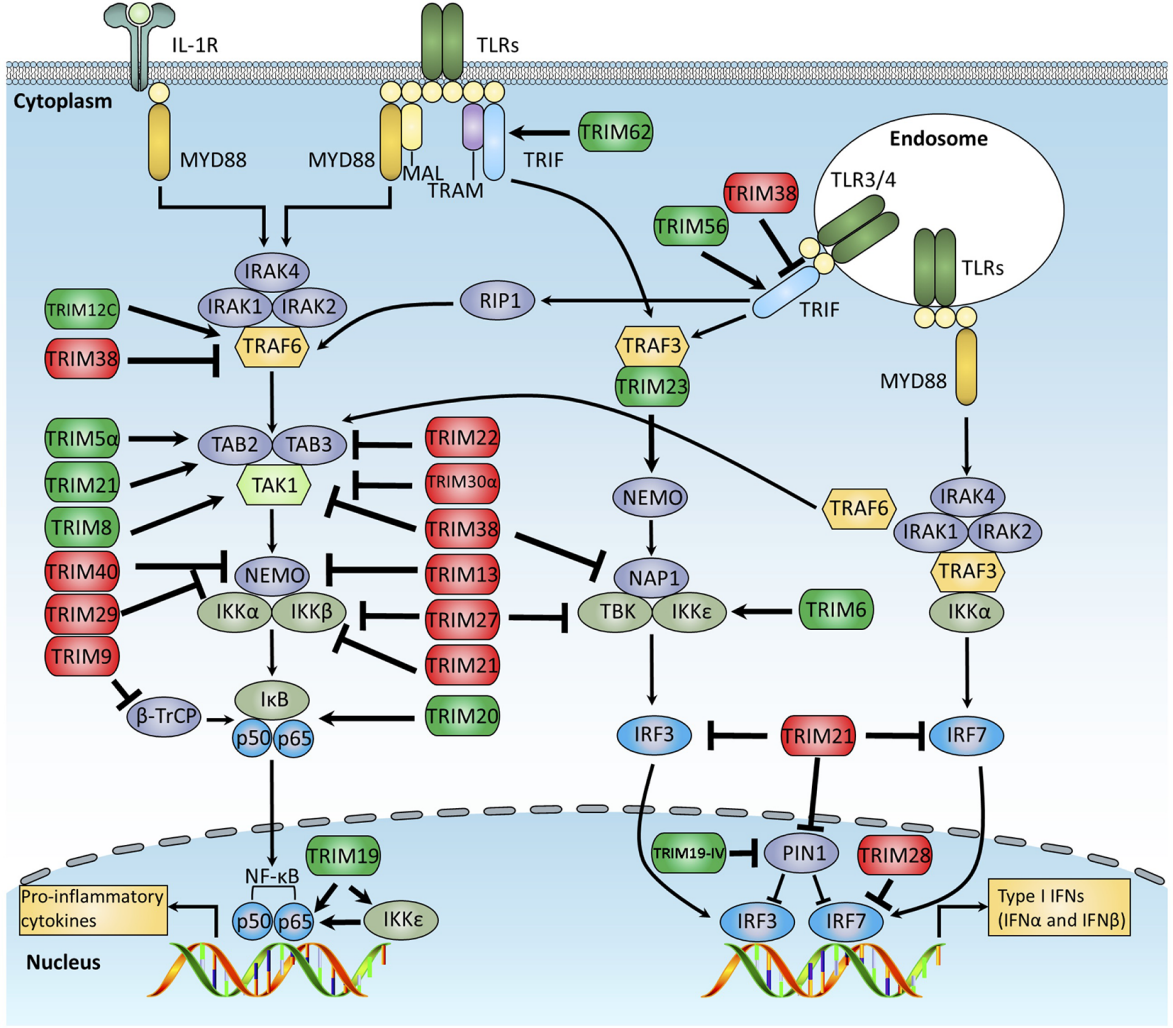
Schematic diagram of TRIM-mediated regulation of the TLR signaling pathway. The TLR signaling is divided into two distinct pathways: the MyD88- and TRIF-dependent pathways. In the MyD88-dependent pathway, activated MyD88 recruits and further activates the IRAK1 kinase complex, which then recruits TRAF6, an E3 ligase that undergoes auto-ubiquitination and mediates the recruitment and auto-phosphorylation of TAK1 complex. Activated TAK1 promotes IκBα phosphorylation, which leads to the degradation of IκBα, and releases NF-κB p50/p65 subunits into nuclear, promote the production of a subset of proinflammatory cytokines. In the TRIF-dependent pathway, activated TRIF recruits TRAF3, an E3 ligase that mediates the activation of TBK1, leading to the phosphorylation and dimerization of IRF3 to promote the production of IFN-I. This figure overviews the known TRIM proteins that regulate the TLR signaling pathway, in which the TRIMs promoting TLR signaling are indicated in green, whereas those suppressing TLR signaling are indicated in red.

There are at least 13 TLRs (10 in human and 12 in mouse) that sense a broad range of PAMPs derived from various pathogens, including viruses, bacteria, fungi, and parasites (1, 3). When stimulated by PAMPs, TLRs transduce signal cascades through the Toll-IL-1-receptor (TIR) domain–containing adaptor proteins, which are roughly divided into two distinct pathways: the myeloid differentiation primary response protein 88 (MyD88)-dependent and the TIR domain-containing adaptor protein inducing IFNβ (TRIF)-dependent signaling pathways. MyD88 is involved in all TLR pathways except for TLR3, whereas TRIF is involved in both TLR3 and TLR4 pathways. By recruiting a series of adaptor proteins and kinases, TLR signal cascades finally trigger the activation of different transcriptional factors, including NF-κB, AP1, and IFN regulatory factors (IRFs), which orchestrate to induce expression of many genes, such as proinflammatory cytokines, chemokines, and type I IFNs. A growing body of evidence indicates the crucial roles of TRIM proteins in both TRIF- and MyD88-dependent pathways (**Figure 2**).

### TRIF-Dependent Signal Pathway

TRIF interacts with receptor interacting protein kinase 1 (RIP1) and TNF receptor-associated factor 3 (TRAF3) to activate distinct signaling pathways. In the TRIF-RIP1 axis, RIP1 interacts with and activates the TGF-β activated kinase 1 (TAK1) complex, leading to the activation of NF-κB and MAPK signaling pathways and inflammatory cytokine production. In contrast, in the TRIF-TRAF3 axis, TRAF3 undergoes self-ubiquitination and mediates the recruitment and activation of IκBα kinase (IKK)–associated kinases TANK-binding kinase 1 (TBK1)/IKKϵ, resulting in the phosphorylation and transcriptional activation of IRF3/IRF7 (3). Interestingly, TRIM proteins are found to regulate TRIF-mediated signaling by different mechanisms. TRIM56 is demonstrated to participate in TLR3 signaling as a scaffold protein by forming a TRIF-associated complex. Biochemical studies reveal that the ubiquitin E3 ligase activity of TRIM56 is dispensable for TRIM56 to regulate TLR3-mediated signaling, and the C-terminal of TRIM56 that mediates TRIF-TRIM56 interaction is critical for type I IFN production (28). Functions of TRIM56 in intracellular dsDNA exposure and Pestivirus infection have also been studied (discussed below) (75, 117). In contrast to TRIM56, TRIM38 negatively regulates TLR3- and TLR4-mediated type I IFN production by targeting TRIF for degradation; the PRY-SPRY domain of TRIM38 is crucial for the interaction of TRIM38 with TRIF, leading to the K48-linked poly-ubiquitination and subsequent degradation of TRIF in the RING/B-Box domain-dependent manner (26). TRIM38 deficiency in bone marrow–derived macrophages (BMDMs) results in a striking elevation of proinflammatory cytokines and type I IFN production upon LPS and poly (I:C) stimulation. In vitro studies indicate that TRIM38 targets and mediates K48-linked poly-ubiquitination of TRIF at K228, and *in vivo* studies show that mice deficient of TRIM38 are more susceptible to poly (I:C)-, LPS- and *Salmonella typhimurium-*induced death due to the overwhelming inflammatory response, suggesting a negative regulatory role of TRIM38 in the TLR3 and TLR4 signal pathways (27). Additionally, TRIM8 negatively regulates the TRIF-dependent pathway through a different mechanism. It is revealed that TRIM8 catalyzes K6- and K33-linked polyubiquitination of TRIF, resulting in the disruption of TRIF-TBK1 interaction and, thereby, inhibition of the TLR3/4-mediated innate immune response (118). In addition, TRIM32 can induce TRIF degradation by TAX1BP1-dependent autophagy. Deficiency of TRIM32 results in enhanced TLR3/4-mediated immune responses, such as elevated IFN-I and proinflammatory cytokine production (119).

TBK1 and IKKϵ are crucial kinases downstream of the TRIF-dependent signal pathway to activate IFN-I production, the ubiquitination of which are recently suggested to be essential for TRIF signaling activation (120). TRIM27, also named Ret finger protein (RFP), is demonstrated to interact with IKKϵ as well as IKKα and IKKβ (32). Knockdown of TRIM27 *via* RNA interference enhances ISRE and/or NF-κB element activation, suggesting the negative role of TRIM27 in regulating inflammatory and antiviral responses. Interestingly, TRIM6 was recently reported to regulate the IKKϵ-mediated innate immune response. TRIM6 cooperates with the E2 ubiquitin conjugase UbE2K to produce unanchored K48-linked polyubiquitin chains that activate IKKϵ to phosphorylate STAT1 at S708, resulting in a range of IFN-stimulated gene (ISG) expression in response to IFNβ stimulation and subsequent antiviral response both *in vitro* and *in vivo* (104). This study reveals that K48-linked polyubiquitin chains could also have the potential to be involved in kinase activation. TBK1 is another crucial kinase that mediates TRIF and RLR signaling activation; the regulation of TBK1 by TRIM proteins is discussed later.

IRF3 and IRF7 are essential transcription factors to induce IFN-I production, which are also currently reported to be tightly modulated by TRIM proteins. TRIM28, also named KRAB-associated protein 1 (KAP1), acts as the E3 ligase of IRF7 to induce the SUMOylation of IRF7, which suppresses the transcriptional activity of IRF7 and, thus, negatively regulates cellular antiviral immune responses (33). Similarly, TRIM21 interacts with IRF7 and ubiquitinates IFR7 in a dose-dependent manner, which leads to a decrease of IRF7 protein expression level and, thus, inhibition of IFN-I production, suggesting TRIM21 has the potential to protect hosts from overwhelming innate immune response through a negative feedback loop (34). Notably, TRIM21 does not target IRF7 specifically, but promotes IRF3 degradation through the ubiquitination of IRF3, indicating that TRIM21 is a critical negative regulator of the IFN-I signaling pathway (35). In addition to the ubiquitin-proteasome–mediated degradation of IRF3 by TRIM21, a recent report reveals that TRIM21 has the potential to target IRF3 for autophagy-dependent degradation (36, 37). Interestingly, TRIM19IV, which was previously identified to prevent IRF3 degradation through the recruitment of the prolyl isomerase Pin1, was recently demonstrated to associate with activated IRF3 and promote IRF3 degradation through the ubiquitin-proteasome pathway, suggesting different roles of TRIM19IV in the tight regulation of IFN-I production by targeting IRF3 (38, 39). Additionally, TRIM26 is reported to negatively regulate IFNβ production mediated by TLR3/4, RLRs, and DNA sensing pathways. Further study indicates that, upon virus infection, TRIM26 is upregulated significantly and is translocated to the nucleus, in which TRIM26 binds to IRF3 and promotes K48-linked polyubiquitination and subsequent degradation of IRF3, leading to the suppression of the antiviral response (68).

### MyD88-Dependent Signaling Pathway

In the MyD88-mediated signaling pathway, TRAF6 bridges the communication between TLRs and downstream kinases, and TRAF6 ubiquitination is essential for the downstream kinase interactions, including the TAK1 complex. It is also interesting that few TRIM proteins are reported to directly regulate MyD88 although an increasing amount of evidence indicates TRIM proteins regulate the TRAF6-TAK1-IKK axis through different mechanisms. TRIM13 is found to catalyze K29-linked polyubiquitination of TRAF6 and, therefore, promote NF-κB signaling activation in the TLR2-mediated immune response (42), and TRIM38 is reported to bind to TRAF6 and promote K48-linked polyubiquitination of TRAF6 in macrophages, leading to the degradation of TRAF6 and, thus, suppression of NF-κB activation (40).

TAK1 is the crucial kinase that controls the activation of NF-κB in the MyD88 signaling activation, which is also regulated by TRIM proteins. There is a series of *TRIM* genes located at the short arm of human chromosome 11, including *TRIM3*, *TRIM5*, *TRIM6*, *TRIM21*, *TRIM22*, *TRIM34*, *TRIM66*, and *TRIM68* (18), which form a gene cluster and are considered to be the result of evolution to enhance cellular antiviral activities (121–125). TRIM5 is well characterized to be an antiviral effector in the study using a retrovirus-infected monkey model (126). Interestingly, TRIM5 generates free K63-linked polyubiquitin chains by its E3 ubiquitin ligase activity, which cooperates with the E2 conjugating enzymes UEV1A and UBC13, serving as a scaffold to recruit the TAK complex and leading to the activation of TAK1 and downstream transcription factors, such as AP1 and NF-κB (43, 44). Interestingly, TRIM21 promotes TAK1 activation through a similar mechanism by catalyzing free K63-linked polyubiquitin chains (47, 48). TRIM22, which is identified to be an IFN-I-induced TRIM protein, was recently reported to suppress the self-ubiquitination of TRAF6 and interact with TAB2, leading to TAB2 degradation and, thus, the suppression of TRAF6-induced NF-κB signaling activation (46, 127). Different from the human *TRIM5* gene cluster, there are also more than eight *Trim5*-like genes, including *Trim5*, *Trim12*, *Trim30*, encoded in the *Trim5* cluster of mouse chromosome 7 (124, 125). TRIM12c (previously known as TRIM12-2), the mouse homolog of human TRIM5, is found to promote the ubiquitination of TRAF6 and, thus, positively control the IRFs and the NF-κB activation (41). In contrast, TRIM30α, another murine homolog of human TRIM5, negatively regulates LPS-induced NF-κB signaling activation by interacting with the TAK1 complex and promoting TAB2 and TAB3 degradation through a pathway independent of the ubiquitin-proteasome system as well as inhibiting TRAF6 auto-ubiquitination (45). TRIM8 and TRIM38 also regulate the TAK complex. TRIM8 mediates TNFα- and IL-1β-induced NF-κB activation by promoting K63-linked polyubiquitination of TAK1 (99, 128), whereas TRIM38 negatively regulates TNFα- and IL-1β-induced signaling pathways by mediating TAB2/3 degradation in a lysosomal-dependent manner (27, 100).

The IKK complex, consisting of NEMO (IKKγ), IKKα, and IKKβ, is downstream of TAK1, which plays essential roles in activating NF-κB and proinflammatory cytokine production. TRIM23 interacts with both TRAF3 and NEMO and mediates K27-linked polyubiquitin conjugation of NEMO, which is necessary for both TLR3- and RIG-I-mediated antiviral inflammatory and IFN production, and this seems to be a parallel regulation of TRAF6-mediated, K63-linked, and linear ubiquitin chain assembly complex (LUBAC)-mediated linear ubiquitination of NEMO (29, 129). A recent study reveals that TRIM23 has dual E3 ubiquitin ligase and GTPase activities (130). TRIM23 can catalyze K27-linked auto-ubiquitination, which is essential for GTP hydrolase activity. Furthermore, the GTP hydrolase activity of TRIM23 is crucial for the activation of TBK1- and p62-mediated selective autophagy, suggesting there is another mechanism for TRIM23-mediated antiviral response. Another study proposes that TRIM23 is involved in human cytomegalovirus (HCMV)-induced NF-κB activation through the regulation of TRAF6 autoubiquitination in the presence of UL144, one of the HCMV gene products (30). However, the detailed mechanism for TRIM23 in the regulation of NF-κB signaling activation still needs to be further studied.

It should be noted that different types of ubiquitin chains and different ubiquitin sites of NEMO determine different directions of the signaling cascades in response to different stimulations. For example, K63-linked and linear ubiquitination of NEMO is essential for TNFα receptor–mediated NF-κB activation, whereas K27-linked ubiquitination of NEMO plays crucial roles in Sendai virus (SeV)-induced NF-κB activation (29). In addition, NEMO can also undergo K48-linked poly-ubiquitination, which negatively regulates the NF-κB pathway. It is demonstrated that TRIM29 functions in macrophages to respond to bacterial and viral infections in the respiratory tract. Deletion of *Trim29* enhances the production of IFN-I and proinflammatory cytokines in macrophages and, thus, protects mice from influenza virus infection. Mechanistically, TRIM29 is studied to interact with NEMO directly and mediates K48-linked ubiquitination and subsequent proteolytic degradation of NEMO, which suppresses the cellular antiviral immune response significantly (31). Furthermore, TRIM40 is reported to suppress the NF-κB signaling activation by binding to the ubiquitin-like protein NEDD8 and mediating the neddylation of NEMO (49), and TRIM13 is shown to interact with NEMO and mediate its ubiquitination, resulting in the turnover of NEMO and suppression of TNFα-induced NF-κB activation (101).

In addition to NEMO, additional subunits of the IKK complex are also studied to be modulated by TRIM proteins. It is reported that TRIM21 interacts with IKKβ and catalyzes monoubiquitin of IKKβ in cooperation with the E2-conjugating enzyme UbcH5B (50, 51). The monoubiquitinated IKKβ is consequently sequestered in autophagosome and degraded through autophagolysosome, which leads to the suppression of NF-κB activation induced by the HTLV-I viral protein Tax. Notably, TRIM proteins also regulate NF-κB signaling downstream of the IKK complex. Among these TRIMs, TRIM20, also named pyrin, an essential protein in familial Mediterranean fever (FMF, a recessively inherited systemic autoinflammatory disorder), is reported to undergo cleavage by caspase-1. Upon cleavage, the pyrin domain of TRIM20 is released to interact with IκBα and p65, leading to the IκBα degradation and translocation of p65 into the nucleus, and finally, promoting NF-κB activation (54). Another study reveals that TRIM19 directly interacts with RelA/p50 to prevent the binding of RelA/p50 to its target genes (52). Interestingly, TRIM9 interacts with the WD-repeat region of β-TrCP, which plays pivotal roles in both the canonical and noncanonical NF-κB pathways by regulating IκBα and precursor p100 for proteasomal degradation and processing, respectively (53). This interaction between TRIM9 and β-TrCP finally stabilizes β-TrCP, thus inhibiting both canonical and noncanonical NF-κB signaling pathways. Considering TRIM regulates NF-κB signaling activation at multiple levels, it is important to further illuminate the regulating network of TRIMs in the NF-κB signaling pathway.

## Trims Function in RLR-Mediated Innate Immune Response

RLRs are the crucial cytoplasmic PRRs that are responsible for detecting RNA virus infections (1, 4, 131). RLRs are family of DExD/H box RNA helicases and are composed of three members: retinoic acid-inducible gene I (RIG-I), which recognizes short viral RNA with a 5’-triphosphate group; melanoma differentiation-associated gene 5 (MDA5), which prefers to sense long viral dsRNA; and laboratory of genetics and physiology (LGP2), which plays important roles in regulating RIG-I and MDA5 pathways. Upon stimulation, both RIG-I and MDA5 trigger signal transduction through the activation of mitochondrial antiviral signaling protein (MAVS, also named IPS1, VISA, or Cardif), which leads to activation of TBK1 and IKKϵ protein kinases and finally activates NF-κB and IRFs to induce the production of proinflammatory cytokines and IFN-I (132).

**Figure 3:**
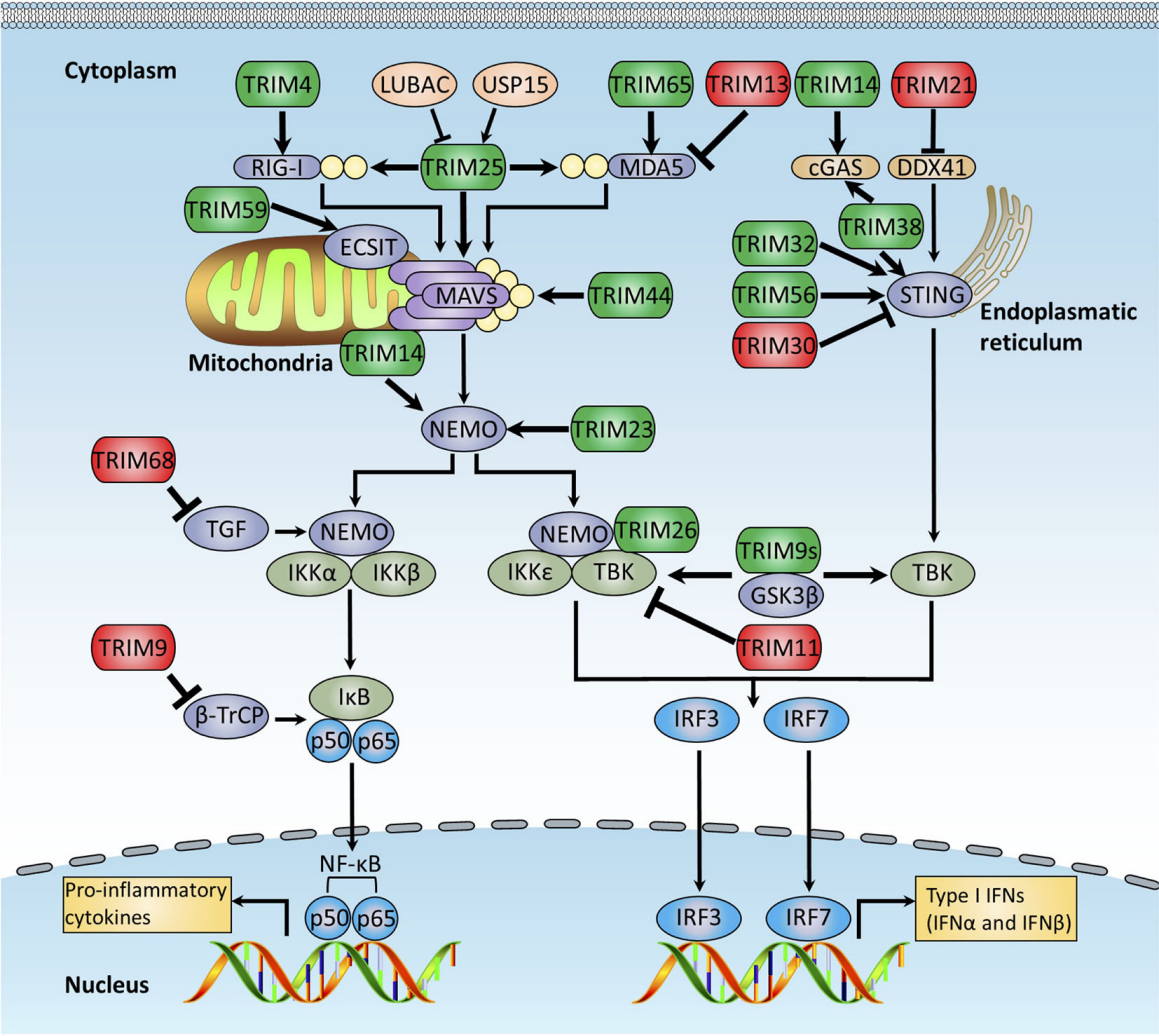
TRIMs regulate RLRs and cytosolic DNA-sensing receptor pathways. The RLRs and cytosolic DNA-sensing receptor pathways regulated by TRIM proteins. RLRs are crucial cytoplasmic PRRs that are responsible for recognition of RNA virus infections, whereas cytosolic DNA-sensing receptors recognize host and microbial DNA in the cytosolic. They both finally activate NF-κB and IRFs to induce the production of proinflammatory cytokines and type I IFNs. This figure illustrates TRIM proteins that modulate RLRs and cytosolic DNA-sensing receptor signaling pathways, and the details of the modulation mechanisms are described in the text. TRIMs promoting RLRs and cytosolic DNA-sensing signaling pathways are indicated in green, whereas these exerting the opposite functions are indicated in red.

Recent studies reveal that the TRIM proteins play crucial roles in regulating the RLR-mediated antiviral response (**Figure 3**). As an E3 ligase of RIG-I, TRIM25 is identified to mediate robust K63-linked polyubiquitination of RIG-I at K172 within the CARD domain, which is required for the formation of the RIG-MAVS complex. The SPRY domain of TRIM25 is found to be responsible for the interaction between TRIM25 and RIG-I (55, 133). Further study indicates that this procedure is a two-step process: TRIM25 first binds to the first CARD domain of RIG-I at T55 and subsequently ubiquitinates its second CARD domain at K172. The T55 in the first CARD domain is critical for the recruitment of TRIM25 to RIG-I, and the K63-linked polyubiquitination at K172 promotes the interaction between RIG-I and MAVS (134). This result also explains why the hepatocyte cell line Huh7.5 carrying the T55I mutation within the first CARD domain of RIG-I dominantly supports hepatitis C virus (HCV) replication (135). The unanchored polyubiquitin chains mediated by TRIM25 could activate the RIG-I pathway. In a cell-free system that mimics viral infection of the intact cell, RIG-I is activated by incubating with both unanchored K63-linked ubiquitin chains and synthesized RNA, leading to the dimerization of IRF3, suggesting that binding of unanchored polyubiquitin chains but not conjugating of polyubiquitin chains is required for RIG-I to activate the cellular antiviral immune response (56). Strikingly, removal of polyubiquitin chains from RIG-I by viral OTU (vOTU), a deubiquitinating enzyme that can cleave both conjugated and unanchored polyubiquitin chains from target proteins, retains the ability to activate IRF3, suggesting that polyubiquitin chains function as functional molecules to activate RIG-I. In support of this notion, the T55I and K172R mutants of RIG-I could not bind to K63-ubiquitin chains or activate IRF3. Despite the role of unanchored polyubiquitin chains in the activation of RIG-I, TRIM25 is needed for the ubiquitination and subsequent activation of RIG-I as depletion of TRIM25 significantly suppresses RIG-I-mediated IRF3 activation due to decreasing the production of free endogenous polyubiquitin chain production (56). Moreover, TRIM25 was recently demonstrated to ubiquitinate MAVS and promote its degradation *via* the proteasome-dependent way, which leads to the release of activated signaling complex from mitochondria into cytosol and activation of downstream signaling, including NEMO and TBK1 (57). Additionally, TRIM25 modulates MDA5-MAVS signaling axis–mediated NF-κB activation through TRAF6 (136).

Interestingly, the TRIM25 protein level was also found to be tightly regulated in a ubiquitin-dependent manner. The linear ubiquitination of TRIM25 mediated by LUBAC (containing two E3 ligases, HOIL-1L and HOIP), leads to the degradation of TRIM25, which results in impaired IRF activation and IFN-I production (137). Furthermore, the NZF domain of HOIL-1L is identified to compete with TRIM25 to bind to RIG-I, providing another mechanism for LUBAC to negatively regulate TRIM25 function and cellular antiviral immune response. However, the deubiquitinase USP15 is reported to hydrolyze K48-linked polyubiquitin chains from TRIM25 mediated by LUBAC, which restores antiviral immune response (138). Because LUBAC also mediates linear ubiquitination of TRIM25, it is still not clear whether USP15 affects the linear ubiquitination of TRIM25 mediated by LUBAC.

Given the critical functions of TRIM25 in the antiviral immune response, it is not surprising that many feedback mechanisms are developed by viruses to counteract the antiviral activity of TRIM25. It is demonstrated that SeV infection induces robust expression of a splice variant of RIG-I, which carries a short deletion (amino acid 36-80) in the first CARD domain within the RIG-I molecule and, thus, loses the ability to recruit TRIM25, leading to the disruption of the interaction between TRIM25 and RIG-I and the suppression of downstream antiviral signaling activation (134). Additionally, Kaposi’s sarcoma-associated herpesvirus (KSHV)–encoded protein ORF64 is found to specifically inhibit TRIM25-mediated K63-linked polyubiquitination of RIG-I and then suppress the activation of the RIG-I signaling pathway (139). Additionally, influenza A virus nonstructural protein 1 (NS1) specifically targets TRIM25 and interferes with the interaction between TRIM25 and RIG-I, leading to attenuated IFN-I production and an antiviral immune response (140). Recent studies reveal the structure of TRIM25 protein and identify a putative binding pocket in the TRIM25 SPRY domain, which is critical for the interaction between TRIM25 and RIG-I (141). This study highlights that small molecular compounds, which target the binding pocket of TRIM25, have the potential to be promising therapeutic drugs to inhibit activation of the RIG-I signaling pathway in some autoimmune diseases or acute hypersensitive diseases (142). In addition to TRIM25, TRIM4 is another well-studied regulator of RIG-I through K63-linked polyubiquitination of RIG-I, which positively regulates virus-induced activation of NF-κB and IRF3 as well as IFNβ production. However, TRIM4 is found to compete with TRIM25 to bind to the RIG-I molecule, suggesting that they may play redundant roles in regulating RIG-I signaling activation (58).

Like RIG-I, the MDA5-mediated signaling pathway is also modulated by the TRIM proteins. TRIM65 is found to enhance MDA5-mediated type I IFN production through interaction with MDA5. It is interesting that the *Salmonella*-derived SopA, a HECT-type E3 ligase, is reported to modulate the RLR signaling pathway by targeting TRIM56 and TRIM65 and enhancing TRIM56 and TRIM65-mediated MDA5-induced type I IFN production, suggesting that *Salmonella* can regulate cellular inflammatory response through RLR signaling (59). A recent study also reveals TRIM65 can directly catalyze K63-linked ubiquitination of MDA5, which is essential for its antiviral activity (60). However, TRIM13 was recently found to interact with MDA5 directly and negatively modulate MDA5-mediated IFN-I production *in vitro*. The deficiency of TRIM13 in mice enhances the product of IFN-I in response to encephalomyocarditis virus (EMCV) challenge and prolonged mice survival (61).

As the most critical adaptor protein in RIG-I and MDA5-mediated signaling pathways, MAVS is regulated by several TRIM proteins. TRIM14, lacking the RING domain and E3 ligase activity, is found to interact with MAVS on the outer membrane of mitochondria directly, suggesting TRIM14 as a crucial mitochondria adaptor in RLR-mediated signaling. Further study reveals that viral infection promotes MVAS-TRIM14 complex formation, and TRIM14 then recruits NEMO to the MAVS signalosome *via* K63-linked polyubiquitin chains, resulting in the activation of both NF-κB and IRF3 signaling pathways (62). Similarly, TRIM44 is also indicated to interact with MAVS and prevent MAVS ubiquitination and degradation, thus enhancing antiviral response (63). Also, a recent study reveals that TRIM31 catalyzes K63-linked polyubiquitination of MAVS, leading to MAVS aggregation and activation (64). Furthermore, TRIM21 interacts with MAVS and induces K27-linked polyubiquitination of MAVS, which then promotes the recruitment of TBK1 to MAVS and inhibits viral infection (143). TRIM29 is recently reported to negatively control the immune response against dsRNA virus infection. Deletion of TRIM29 conferred mice more resistant to dsRNA virus reovirus infection. Mechanistically, TRIM29 interacts with MAVS and induces K11-linked polyubiquitination and subsequent proteasomal degradation of MAVS (144).

Activation of MAVS thereby recruits the IKK complex as well as the noncanonical IKK related kinases, IKKϵ and TBK1, *via* similar mechanisms seen in TLR-mediated signaling cascades. TRIM proteins have also been studied to play important roles in regulating the RLR signaling pathway. Upon viral infection, TRIM9 short isoform undergoes K63-linked auto-polyubiquitination, which, in turn, serves as a platform to recruit GSK3β to TBK1 and promotes the phosphorylation of TBK1 in a GSK3β-dependent manner, resulting in IRF3 activation, instead of inhibiting NF-κB mediated proinflammatory cytokine production (65). It is reported that TRIM26 negatively regulates antiviral immune response by targeting IRF3 (68), and in a recent study, TRIM26 functions as a positive regulator in RNA virus-induced antiviral innate immunity. Upon RNA virus infection, TRIM26 undergoes autoubiquitination. Subsequently, the ubiquitinated TRIM26 interacts with NEMO and then bridges the interaction between NEMO and TBK1, which promotes the recruitment of TBK1 to MAVS, leading to activation of TBK1 and the downstream signaling pathway (67). Probably, these apparent discrepancies of TRIM26 in the regulation of virus-induced RLR signaling activation might be due to the different cell types used in those studies. An additional study demonstrates that TRIM38 negatively regulates RIG-I and TLR3/4 signaling pathways through the interaction with and mediation of K48-linked polyubiquitination and subsequent proteasomal degradation of NAP1 (66), which is required for both RIG-I and TLR3/4-mediated IFNβ production by activating TBK1.

Additionally, TRIM11 and TRIM68 are reported to regulate antiviral immune responses by targeting TBK1 negatively and the TRK-fused gene (TFG), which is an activator of NF-κB, by interacting with NEMO and TANK, respectively, thus inhibiting IFN-I and inflammatory cytokine production (69, 70). Notably, the activation of the RLR-mediated antiviral immune response is tightly regulated by TRIM proteins. A recent study investigates all known human TRIM proteins and demonstrates that half of them have the potential to enhance innate immune response (20). This study also indicates that 19 TRIM proteins out of the explored TRIMs are involved in the RIG-I-mediated signaling pathway, providing a comprehensive resource to further investigate the functions of the TRIM proteins in the RLR-mediated signaling pathway.

## TRIMs Function in the Cytosolic DNA-Sensing Receptor–Mediated Innate Immune Response

The cytosolic DNA-sensing receptors are important PRRs that recognize host and microbial DNA in the cytosolic. Recently, more than 10 DNA-sensing receptors, including cGAMP synthetase (cGAS), IFN-inducible protein 16 (IFI16), and DEAD/H BOX 41 (DDX41), were identified (2, 145). Most of them are found to employ stimulator of IFN genes (STING, also named MITA), a transmembrane protein localized on the endoplasmic reticulum (ER), as the adaptor protein to recruit TBK1 and initiate proinflammatory cytokine and IFN-I production, facilitating the elimination of invading pathogens.

It was recently studied that the TRIM proteins play crucial roles in regulating cytosolic DNA-sensing receptor–mediated innate immune response (**Figure 3**). A recent study reports that TRIM14 interacts with cGAS and then recruits USP15 to cleave the polyubiquitin chains on cGAS, resulting in the inhibition of cGAS degradation and facilitating antiviral innate immune response (73). TRIM38 has the potential to prevent cGAS polyubiquitination and degradation by mediating the SUMOylation of cGAS. TRIM38 is also found to SUMOylate STING during the early stage of virus infection, which promotes STING protein stability and activation of the downstream signaling pathway (74). Upon the invasion of antibody-opsonized virus, TRIM21 intercepts virions immediately and promotes viral genome exposure to both the DNA sensor cGAS and RNA senor RIG-I in the antibody-dependent manner, thus leading to rapid inflammatory cytokine production and virus clearance (146). However, the SPRY domain of TRIM21 is reported to interact with the DEAD domain of DDX41, leading to the K48-linked ubiquitination and subsequent degradation of DDX41 (71), suggesting differential roles of TRIM21 in antiviral immune response depending on different sensors. The function of TRIM21 as a negative regulator of the DNA sensor also indicates that TRIM21 may function not only in antiviral response, but also in autoimmune diseases, such as systemic lupus erythematosus.

TRIM56 is constitutively expressed in most tissues, and its expression is found to be upregulated upon IFN-I stimulation and virus infection (117). Further studies reveal that IFN-I-induced TRIM56 expression restricts virus infection significantly through its interaction with STING, which promotes K63-linked ubiquitination of STING and leads to the recruitment of TBK1 to increase IFN-β production (75). Similarly, TRIM32, localized on mitochondria and endoplasmic reticulum, interacts with STING to promote antiviral immune response (76). Overexpression of TRIM32 promotes virus-induced IFNβ production, whereas knockdown of TRIM32 leads to the opposite outcomes. However, another study suggests that TRIM56 and TRIM32 might not be the direct E3 ubiquitin ligases of STING, and additional proteins in the STING complex are responsible for the ubiquitination of STING (147). Indeed, a recent study reveals that TRIM56 could directly interact with cGAS and promote K27-linked mono-ubiquitination of cGAS at K335, leading to the enhancement of cGAS dimerization, DNA-binding activity, cGAMP production, and finally, increase of anti-DNA virus response (148).

Contrary to TRIM32 and TRIM56, TRIM30α is reported to be a negative-feedback regulator of STING (77). Upon herpes simplex virus type 1 (HSV-1) infection, TRIM30α expression is upregulated, and then TRIM30α interacts with STING and promotes K48-linked ubiquitination of STING at K275, resulting in its subsequent proteasome-dependent degradation and attenuated antiviral immune response. Consistently, knockdown or genetic deficiency of TRIM30α enhances both intrinsic DNA and DNA virus-induced IL-6 and IFN-I production significantly. Also, *Trim30α* ^-/-^ conferred mice more resistant to DNA virus infection. TRIM29 also negatively regulates antiviral immune response by targeting STING for K48-linked polyubiquitination and subsequent proteasomal degradation. Similar to TRIM30α, TRIM29 expression is robustly induced by cytosolic DNA stimulation in dendritic cells and macrophages, which leads to enhanced proinflammatory cytokine and IFN-I production (149, 150).

## TRIMs Function in NLRs and Inflammasome-Mediated Innate Immune Response

The NLRs are intracellular PRRs sensing PAMPs or DAMPs associated with cell stress (151). Upon stimulation, most NLRs, including NLRP1 (NOD-, LRR- and pyrin domain-containing 1) and NLRP3, recruit an adaptor protein, the apoptosis-associated speck-like protein containing a CARD domain (ASC), and the effector protein, pro-caspase-1, forming a large complex known as an inflammasome, resulting in the cleavage and activation of pro-caspase-1. Activated caspase-1 further cleaves IL-1β and IL-18 precursors into functional forms, which are subsequently secreted out of the cell to induce inflammatory responses.

**Figure 4:**
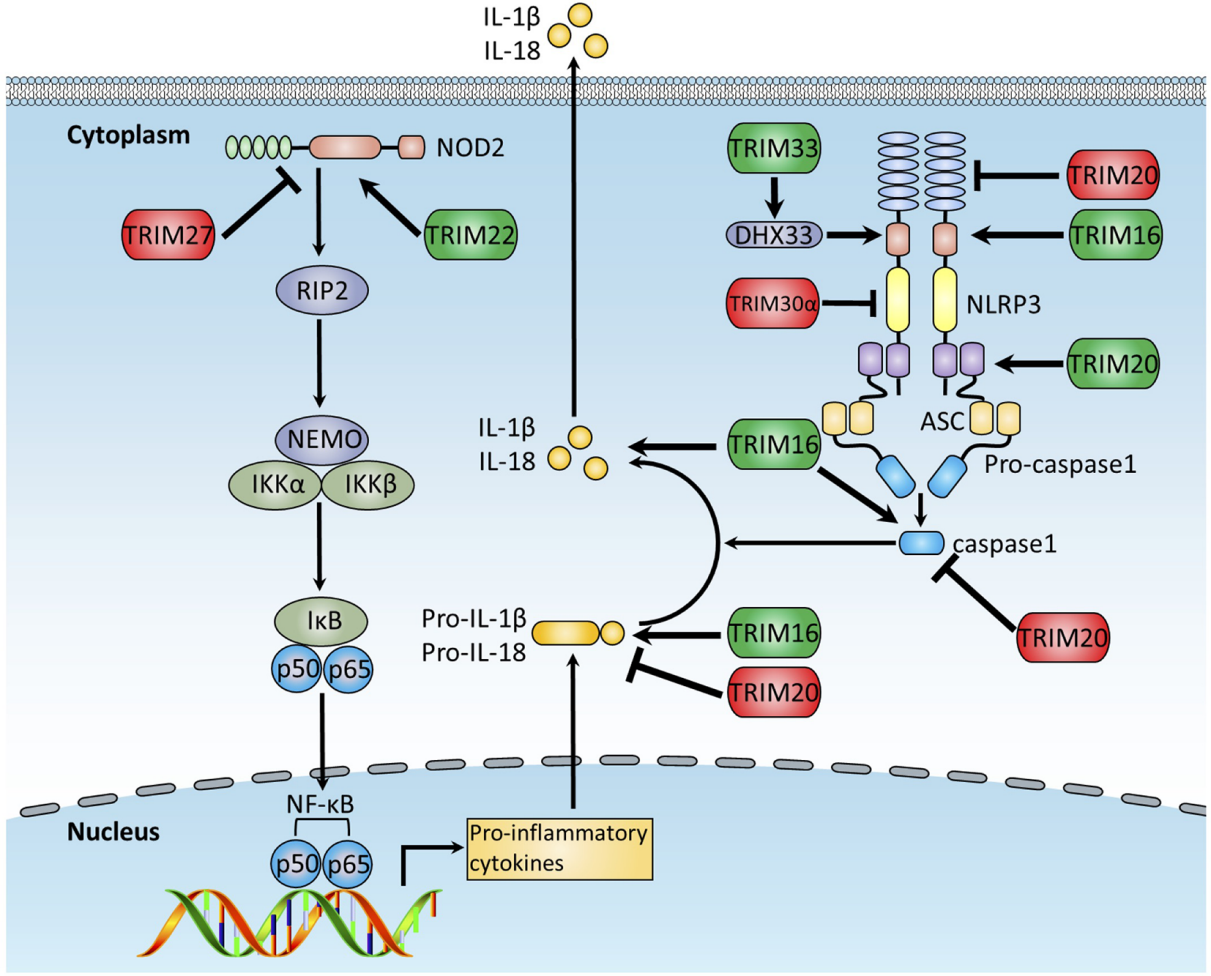
The function of TRIMs in NLRs and inflammasome pathways. The NLR and inflammasome pathways are intracellular PRRs sensing PAMPs or DAMPs associated with cell stress. Activated inflammasome activates pro-caspase-1 and cleaves IL-1β and IL-18 precursors into the functional forms, which are subsequently secreted out of the cell to induce inflammatory responses. This figure illustrates TRIM proteins regulating NLRs and inflammasome pathways. TRIMs promoting NLRs and inflammasome pathways are indicated in green, whereas these exerting the opposite functions are indicated in red.

NLRs are well studied for their crucial functions in inflammasome signaling, and it is reported that TRIM proteins play important roles in regulating the inflammasome signaling pathway (**Figure 4**). TRIM20 is involved in a series of autoimmune diseases, including FMF and PAPA syndrome (pyogenic arthritis, pyoderma gangrenosum, and acne syndrome) by modulating NLRP1 and NLRP3 inflammasomes through interacting with the vital adaptor protein in the inflammasome signal pathway and being part of the inflammasome (116, 152). Previous studies indicate that TRIM20 either positively (81, 153–155) or negatively (78–80) regulates the inflammasome signaling pathway. One potential explanation for the functional discrepancies of TRIM20 is that full-length TRIM20 inhibits inflammasome signaling activation, and some stimuli trigger the release of the PYD domain of TRIM20, which then interacts with ASC and induces ASC oligomerization, leading to the activation of inflammasome (81). In agreement with this notion, macrophages from genetically modified mice carrying null *Trim20* alleles were found to produce more IL-1β than wild-type mice in response to a spectrum of inflammatory stimuli (80). Additional studies indicate that the SPRY domain of TRIM20 negatively regulates inflammasome activity through the interaction with NLRP3, caspase-1, and pro-IL-1β (78, 79). Indeed, TRIM20 knock-in mice harboring the mutant of the human SPRY domain show spontaneous development of bone marrow-dependent inflammation, which is similar to human FMF symptoms, mediated by ASC-dependent but NLRP3-independent production of IL-1β from bone marrow-derived cells (156). Additionally, TRIM20 was recently reported to negatively regulate inflammasome activation through targeting NLRP1, NLRP3, and pro-caspase-1 for precise autophagic degradation (36, 157).

TRIM16, a RING-absent TRIM protein member similar to TRIM20, is demonstrated to promote IL-1β secretion through the binding to NLRP1, caspase-1, and pro-IL-1β (82), and TRIM30 was found to negatively regulate NLRP3-mediated caspase-1 activation and IL-1β production in response to a range of NLRP3 inflammasome agonists, including monosodium urate, silica, ATP, and nigericin (83). Overexpression of TRIM30 decreases reactive oxygen species (ROS) production significantly, whereas knockdown of TRIM30 increases ROS production. However, attenuation of ROS through antioxidant treatment eliminates amplified IL-1β production in TRIM30-knockdown cells, suggesting that TRIM30 negatively regulates NLRP3 inflammasome-mediated IL-1β production in a ROS-dependent manner (83). Additionally, TRIM31, a positive regulator of RLRs-mediated type I IFN signaling (64), is demonstrated to negatively regulate NLRP3 inflammasome signaling by promoting K48-linked polyubiquitination and subsequent proteasomal degradation of NLRP3 (84).

TRIM proteins also regulate the upstream proteins of the inflammasome. DHX33 is a cytosolic dsRNA sensor to activate NLRP3 inflammasome signaling upon dsRNA stimulation (158). TRIM33 can directly bind to DHX33 and, thus, induce K63-linked ubiquitination of DHX33 at K218, which is crucial for the formation of the DHX33-NLRP3 inflammasome complex, thereby leading to NLRP3 inflammasome signaling activation (85). Additionally, TRIM22 interacts with NOD2 and mediates its K63-linked ubiquitination, which is crucial for proinflammatory response and very early onset of inflammatory bowel disease (159). On the contrary, TRIM27 is reported to bind directly to and mediate K48-linked ubiquitination and subsequent proteasomal degradation of NOD2, leading to the suppression of NOD2-mediated inflammatory response, suggesting TRIM27 as a potential therapeutic target for NOD2-associated inflammatory diseases (86).

## TRIMs Regulate T Cell Functions

The crucial roles of TRIM proteins in the innate immune response to pathogen invasions have been intensively studied. Interestingly, the involvement of TRIM proteins in adaptive immune response, especially in the regulation of T cell function, is also currently being explored.

The full activation of T cells requires two distinct signals, the stimulation of TCRs by the MHC-peptide complex and costimulatory molecules, especially CD28, which finally initiates the downstream signaling cascades and of T cell activation and proliferation. Tyrosine kinase LCK is the first activated kinase upon TCR stimulation, which, in turn, phosphorylates the CD3ζ chain and ZAP70. Activated ZAP70 induces the activation of several substrates, including LAT and SLP-76, to transduce the signal to PLCγ-1, and downstream PI3K-AKT, MAPK, NF-κB, and calcium-dependent signaling pathways. All these signaling events finally result in the boost of transcriptional activity of NF-κB, AP1, and NF-AT, which induce gene expression for T cell activation, proliferation, differentiation, and survival (10). Because T cells serve as central components of adaptive immunity, it is not surprising that tight regulations are involved in T cell activation and functions. Although many E3 ubiquitin ligases so far have been studied for their important roles in T cell–mediated immune response and tolerance, including c-Cbl, GRIAL, and ITCH, the functions of TRIM proteins in T cell signaling are yet largely unknown.

The class II phosphatidylinositol 3 kinase C2β (PI3KC2β), which is activated by TCR stimulation, is required for KCa3.1 channel-mediated Ca2^+^ influx and subsequent activation of CD4^+^ T cells. It is reported that TRIM27 interacts with class II PI3KC2β and mediates K48-linked polyubiquitination of PI3KC2β, resulting in the inhibition of PI3K activity and, thus, suppression of T cell activation. Because the KCa3.1 channel is predominated the K^+^ channel in Th0, Th1, and Th2 cells, the deficiency of TRIM27 in T cells exhibits an elevated KCa3.1 channel activity, Ca^2+^ influx, and TCR-mediated cytokine production and proliferation in Jurkat cells, primary human and mouse T cells, and differentiated mouse Th1 and Th2 cells (88). However, TRIM27 seems to be dispensable for the differentiation of CD4^+^ T cells into Th1 and Th2 cells testified by comparable T-bet and GATA3 expression levels, respectively, in WT and *Trim27* ^-/-^ Th1 and Th2 cells.

It is interesting to note that several TRIM proteins play essential roles in both innate and adaptive immunity. Among these TRIMs, TRIM30α, negatively regulating TLR-mediated NF-κB activation by targeting the TAK1/TAB2/TAB3 complex (37), was recently found to be involved in the regulation of T cell functions. Deficiency of TRIM30 has no effect on T cell development and immune homeostasis in young mice, and deficiency of TRIM30 in aged mice exhibits augmented CD4/CD8 T cell ratio, and *Trim30* ^-/-^ CD4 T cells show enhanced cell proliferation upon TCR stimulation both *in vivo* and *in vitro* compared with wild-type T cells. However, despite enhanced cell proliferation, *Trim30* ^-/-^ CD4 T cell activation is compromised upon stimulation, suggested by inhibited NF-κB activation and decreased IL-2 production compared with wild-type cells, indicating that TRIM30 has distinct roles in regulating T cell proliferation and activation (89).

TRIMs also play crucial roles in regulating T cell homeostasis and differentiation. Compared with naïve T cells, the expression of TRIM24 is found to be increased in Th2 cells that are considered to be associated with the pathological progress of allergic asthma (90, 160). Further studies indicate that T cell–intrinsic TRIM24 is responsible for house dust mite (HDM)–induced airway allergy and anti-helminth immunity. However, TRIM24 deficiency in T cells downregulates IL-1R expression significantly and, thus, dampens IL-1-induced transcriptome expression both *in vivo* and *in vitro*, which thereby diminishes IL-1β-mediated airway allergy (90). Additionally, TRIM24 promotes a range of cytokine and chemokine production and the expression of their receptors in T cells, suggesting TRIM24 functions at multiple levels to induce airway allergy. Contrary to TRIM24, TRIM32 is reported to negatively regulate Th2-mediated allergic response. Deficiency of TRIM32 in mice displays as an atopic dermatitis-like inflammatory skin phenotype in response to imiquimod (IMQ) treatment and an increase of cytokine and chemokine expression by Th2 cells (91).

Despite the functions of TRIM21 in innate immunity, overexpression of TRIM21 in Jurkat cells results in increased IL-2 production following CD28 stimulation, whereas knockdown of TRIM21 in Jurkat cells leads to compromised IL-2 production following T cell activation, suggesting that TRIM21 is involved in T cell activation (92). However, a recent study demonstrates that *Trim21* ^-/-^ mice develop severe dermatitis upon tissue injury and, subsequently, several signs of systemic lupus due to hyperactive T cell response (93), suggesting that TRIM21 plays a negative role in T cell-mediated inflammatory response. Furthermore, deficiency of TRIM21 in mice increases several proinflammatory cytokines’ production, including IL-6 and IL-17. Meanwhile, deficiency of IL-23/IL-17 in *Trim21* ^-/-^ mice rescued mice from skin disease and systemic autoimmunity, suggesting TRIM21 negatively regulates systemic autoimmunity through the IL-23-Th17 pathway. One possible explanation for the apparent discrepancy in the two studies could be that TRIM21 functions in distinct roles in primary mouse T cells and the human T cell line (Jurkat). A recent study reveals that TRIM21 expression is increased in monocytes isolated from Behçet’s disease (BD, an autoimmune disease) patients. Monocytes from BD patients facilitate differentiation of Th1 and Th17 when cultured with naïve T cells, whereas knockdown of TRIM21 inhibits Th1 and Th17 differentiation (161), highlighting the synergistic effects of TRIM21 in innate and adaptive immunity.

TRIM28 is highly expressed in both T and B cells; conditional deletion of TRIM28 in T cells leads to autoimmune disorders in mice (96). The deficiency of TRIM28 results in attenuated IL-2 production and a decrease of both CD4^+^ and CD8^+^ T cells in mice. In addition, loss of TRIM28 results in the accumulation of a Th17 autoimmune T cell subset and Foxp3^+^ Treg cell subset because of the suppression of TGF-β3 production and subsequent disruption of the balance of cytokines (96). A molecular mechanism study of the intrinsic role of TRIM28 in Th17 indicates that, upon cytokine stimulation, TRIM28 is recruited to STAT3-occupied genes and regulates epigenetic activation by binding to Th17-specific super-enhancers. Additionally, TRIM28 forms a complex with RORγt and STAT3, which promote RORγt recruited to the promoters of its target cytokine genes (162). Consistently, an additional study demonstrates that T cell–specific deletion of TRIM28 results in the impaired balance of the CD4^+^/CD8^+^ T cell ratio as well as the dramatic expansion of immature thymocytes in mice (163). A mechanism study indicates that TRIM28 functions as a chromatin-remodeling factor to regulate T cell development from an epigenetic perspective (98, 163). In human T cells, TRIM28 plays a role in the suppressive function of Foxp3^+^ Treg through the TRIM28-FIK-Foxp3 interaction (97). Disruption of the TRIM28-FIK-Foxp3 complex in Treg cells abrogates the suppressor activity of Treg and restores the expression of Foxp3-targeted genes. Considering that mouse Foxp3 does not interact with TRIM28 (96, 97), TRIM28 regulates mouse and human Treg in different ways.

## Conclusion and Perspective

In the past decades, the exciting acquisitions of studies exploring the regulatory roles of TRIM proteins in the innate immune response are the involvement of TRIMs in the cross-regulation of innate immune signal pathways, which finely orchestrates host’s innate immune responses against invading microorganisms. For example, TRIM38 negatively regulates TLR3/TLR4-mediated IFN-I production by targeting NAP1 and TRIF for ubiquitin-proteasome-mediated degradation (27, 66); this protein also involves the regulation of TNFα and IL-1β-triggered signal pathways. By targeting TAB2/TAB3 for lysosomal degradation, TRIM38 negatively regulates TNFα and IL-1β-induced NF-κB/MAPK signaling activation and proinflammatory cytokine production (100). Interestingly, TRIM30α negatively regulates TLR-mediated NF-κB activation through a similar mechanism by targeting TAB2/TAB3 for lysosomal degradation (45). More studies about the regulatory network of PRR signaling pathways by TRIM proteins are summarized in **Table 1**.

The outcomes of TRIM proteins in the regulation of differential PRR signal pathways are stimuli and cell-type dependent. TRIM8 enhances TNFα and IL-1β-induced NF-κB activation through promoting K63-linked polyubiquitination of TAK1 (99). However, upon poly(I:C) and LPS stimulation, TRIM8 negatively regulates TLR3/TLR4-mediated proinflammatory cytokine and IFN-I production through the K6/K33-linked polyubiquitination of TRIF, which disrupts the interaction of TRIF with TBK1. TRIM8 deficiency results in enhanced susceptibility to poly(I:C) and LPS-induced death (118). Nevertheless, TRIM8 has been recently proven to be essential for TLR-mediated antiviral innate immune response in plasmacytoid dendritic cells. Further studies illustrate that TRIM8 protects phosphorylated IRF7 from proteasomal degradation by the peptidyl-prolyl isomerase Pin1 in the E3 ubiquitin ligase-independent manner (164). TRIM21 is known to mediate antibody-dependent degradation through the interaction of its PRY/SPRY domain with the Fc domain of antibody (165). Notably, the PRY/SPRY domain of TRIM21 also interacts with the intracellular DNA sensor DDX41, resulting in the K48-linked polyubiquitination and degradation of DDX41 and, thus, impaired innate immune response to intracellular DNA (71). However, TRIM21 facilitates the immune response to RNA virus through interacting with and K27-linked polyubiquitination of MAVS by its PRY/SPRY and RING domain, respectively (143). Therefore, the difference of stimuli and signal pathways should be taken into consideration when the precise functions of TRIMs in innate immune response are studied. From this perspective, regulation of innate immune signal pathways by TRIMs-mediated unconventional posttranslational modifications may be an attractive topic.

Taken together, certain TRIM proteins may have multiple functions, and different TRIMs can coordinate to regulate immune response. Indeed, *TRIM* gene expansions are hugely parallel with the evolution of both innate and adaptive immunity. Therefore, considering the multiple functions of TRIM proteins in both innate and adaptive immunity, it seems clear that TRIM family proteins evolve as regulators to ensure the optimized immune responses to eliminate pathogen infections without autoimmunity.

## Data Availability Statement

The original contributions presented in the study are included in the article; further inquiries can be directed to the corresponding authors.

## Author Contributions 

HH and HZ conceived the paper. WY, ZG, and HH wrote the manuscript. WY and ZG prepared the figures. ZG and HH revised the manuscript. All authors contributed to the article and approved the submitted version.

## Funding

This study was supported by the grants from the Ministry of Science and Technology (the National Key Research and Development Program 2016YFA0502203, 2019YFA0110201, 2019YFA0110203), National Nature Science Foundation of China (91740111, 81871232 and 31870881) and 1.3.5 projection of disciplines of excellence, and National Clinical Research Center for Geriatrics (Z202001001), West China Hospital, Sichuan University.

## Conflict of Interest

The authors declare that the research was conducted in the absence of any commercial or financial relationships that could be construed as a potential conflict of interest.
